# Novel Polymer Sorbents with Imprinted Task-Specific Ionic Liquids for Metal Removal

**DOI:** 10.3390/ma14175008

**Published:** 2021-09-02

**Authors:** Kinga Filipowiak, Patrycja Dudzińska, Karolina Wieszczycka, Tomasz Buchwald, Marek Nowicki, Aneta Lewandowska, Agnieszka Marcinkowska

**Affiliations:** 1 Institute of Chemical Technology and Engineering, Poznan University of Technology, 60-965 Poznan, Poland; kinga.m.filipowiak@doctorate.put.poznan.pl (K.F.); parycja.dudzinska@student.put.poznan.pl (P.D.); aneta.b.lewandowska@doctorate.put.poznan.pl (A.L.); agnieszka.marcinkowska@put.poznan.pl (A.M.); 2 Institute of Materials Research and Quantum Engineering, Poznan University of Technology, 60-965 Poznan, Poland; tomasz.buchwald@put.poznan.pl; 3 Faculty of Materials Engineering and Technical Physics, Poznan University of Technology, 60-965 Poznan, Poland; marek.nowicki@put.poznan.pl

**Keywords:** ionic liquids, sorbents, resins, metals removal, sorption, isotherm, kinetic

## Abstract

In this paper, the potential of novel polymer sorbents with the imprinted IL-functional group for the removal of Cu(II), Cd(II), and Zn(II) from aqueous solutions was investigated by batch mode. The sorbents were fabricated by direct reaction of the prepared polymer matrix (poly(vinylbenzyl chloride-divinylbenzene), VBC, and poly(vinylbenzyl bromide-divinylbenzene), VBBr) with 1-(3- or 4-pyridyl)undecan-1-one and oxime of 1-(3- or 4-pyridyl)undecan-1-one. The Fourier Transform Infrared Spectroscopy (FT-IR), Raman Spectroscopy (Raman), Thermogravimetric Analysis (TG), Differential Scanning Calorimetry (DSC), and Scanning Electron Microscopy (SEM) techniques were used to show functionality and stability of the sorbents. The materials were also characterized by contact-angle goniometry, X-rayphotoelectron spectroscopy (XPS), and Zeta potential analysis. The removal of Cd(II), Cu(II), and Zn(II) was monitored and optimized under the influence of several operational controlling conditions and factors such as pH, shaking time, temperature, initial metal ions concentration, and counter-ions at the functional group. The results obtained confirmed the very high potential of the sorbents; however, the properties depend on the structure of the functional group. The tested sorbents showed fast kinetics, significant capacity at 25 °C (84 mg/g for the Zn(II) sorption with VBC-Ox4.10, 63 mg/g for the Cd(II) sorption with VBBr-Ox3.10, and 69 mg/g for the Cu(II) sorption with VBC-K3.10), and temperature dependence (even 100% increase in capacity values at 45 °C). The selected sorbent can be regenerated without a significant decrease in the metal removal efficiency.

## 1. Introduction

Heavy metals have a wide range of applications in various areas, such as medicine, technology, and the industry [1]. They are also efficient catalysts because of their selectivity and their high activity [2]. Unfortunately, due to the huge technological development, heavy metal ions have become a threat to the natural environment and thus to human health. Pollutants containing ions of heavy metals such as chromium, lead, copper, or cadmium are formed in chemical factories, batch production, mining, and metallurgy [3]. They enter the ground water along with the wastewater from these industries. Heavy metals are highly toxic, not biodegradable, and bioaccumulative. They cause various diseases and disorders, such as cancer [4]. They are very toxic even at a very low concentration. Due to this consideration, many new water treatment processes are being introduced. Conventional methods include precipitation, electrochemical reduction and membrane separation, adsorption, and ion exchange [5]. These methods have limits related to the nature of the metals, their concentration, and the composition of the solution. For example, during precipitation, other contaminants are created due to the use of chemical reagents [6]. The low cost of naturally occurring sorbents encourages researchers to modify their surface, resulting in new sorbents. Adsorption techniques using chelating groups on sorbents’ surface are recently very popular in separation chemistry thanks to the low cost, high adsorption capacity, good regeneration, and good selectivity for certain metal ions [7]. Some of the modified sorbents are silica gel [8], activated carbon [9], zeolites [10], and cellulose derivatives [11]. The functionalization of synthetic sorbents with ionic liquids is an example of process that also fits into the idea of green chemistry. Ionic liquids are alternative media in separation applications as a result of its advantages, such as non-inflammability and non-toxicity [12]. Ionic liquids (IL) such as quaternary pyridinium salts have suitable physicochemical properties for use in the separation processes of organic and inorganic compounds. Particularly high extraction properties and specific mechanism in a liquid–liquid system caused the immobilization of ILs on silica or polymer supports [13,14,15]. By incorporating organic functional groups onto the silica surface, there is an increase in uptake of metals and CO_2_, which improves the process efficiency [16,17,18,19,20], while a combination of polymers and ionic liquids can simultaneously remove both inorganic and organics pollutants from water [21]. The immobilization on polymer supports was performed both in the case of commercial ILs as well as newly designed compounds, although in the first group, mainly impregnation was used. An example is the immobilization of Cyphos IL-101 (tetradecyl(trihexyl)phosphonium chloride) onto Amberlite XAD series sorbents and Pd(II), Fe(III), Hg(II), Au(III), and Pt(IV) sorption from HCl [22,23,24,25]. Our previous studies also confirmed that the functionalization of PS-DVB by the incorporation and encapsulation of ionic liquid extractant [26,27] or by quaternization with IL-precursors to obtain the IL-functionalized polymers enabled us to obtain efficient sorbents [13,28]. The most interesting results were obtained for chromium removal. It was demonstrated that the sorbents functionalized by hydrophilic pyridylketones were responsible not only for adsorption of chromium(VI) but also for a reduction to a less toxic Cr(III). Therefore, the continuation of research, especially in the field of further modification of the functional group and selection of the counter-anion of the quaternary nitrogen in order to obtain novel efficient and stable sorbents, is justified.

Therefore, the goal of this study was to synthesize novel sorbents, copolymers of vinylbenzyl chloride or vinylbenzyl bromide, and divinylbenzene modified by pyridine derivatives consisting of a 3- and 4-position ketone or oxime group for Cu(II), Cd(II), and Zn(II) removal. In the scope of a sorption study, the effect of several parameters including initial pH, metals ions concentration, contact time, and sorption temperature are studied in detail and are discussed to evaluate the efficiency of metal ion removal.

## 2. Materials and Methods

### 2.1. Chemicals

4-(Chloromethyl)styrene (90%, Sigma Aldrich, Darmstadt, Germany) and 1,4-divinylbenzene (85%, Sigma Aldrich, Darmstadt, Germany) were passed through neutral alumina column to remove inhibitor. Tetrahydrofuran (p.a., Sigma Aldrich, Darmstadt, Germany) was distilled over sodium and benzophenone. Benzoyl peroxide (Fluka, Buchs, Switzerland) was dried for 24 h at 60 °C before use. LiBr (99%, anhydrous, Sigma Aldrich, Darmstadt, Germany), toluene (99.9%, POCh, Poznan, Poland), and methanol (99.9%, POCh, Poznan, Poland) were used without purification. 1-(4-pyridyl)undecan-1-one, 1-(3-pyridyl)undecan-1-one, and their oximes were synthesized following a procedure described in [29,30,31]. Copper(II) nitrate (Cu(NO_3_)_2_·6 H_2_O, p.a.) cadmium(II) nitrate (Cd(NO_3_)_2_·4 H_2_O, 99.997%), and zinc(II) nitrate (Zn(NO_3_)_2_·6 H_2_O, p.a.) were purchased from Sigma-Aldrich (Darmstadt, Germany). Standard solutions of Cu(II), Cd(II), and Zn(II) as well as pH buffers were obtained from Merck KGaA (Darmstadt, Germany). HCl (32%), NaOH, and NaCl were of analytical grade and were supplied by Chempur, Poland. Ultra-pure water was obtained by using Arium Pro DI purification system (Sartorius, Göttingen, Germany).

### 2.2. Apparatus

The VBC synthesis, substitution reaction, and functionalization with the pyridine derivatives were confirmed via Raman and FTIR spectroscopy. A Raman spectroscopy analysis was carried out with the use of Renishaw’s inVia Raman System. The Raman spectra of all materials were collected in the spectral range from 3200 to 200 cm^−1^ with lasers emitting 633 nm and 785 nm. Raman map showing the distribution of VBC modified with Ox3.10 was recorded on a 200 μm × 200 μm surface with a 10 μm step size measurement. FT-IR analyses were carried out on a Vertex 70 Spectrometer (Bruker Optics FT-IR) in the range of IR 500–4000 cm^−1^ with a resolution of 2 cm^−1^. The elemental composition of fabricated sorbents was determined using the elemental analyzer FLASH EA 1112 Series of Thermo Electron Corporation.

Thermogravimetric investigations were performed on a TG 209 F3 Tarsus analyzer (NETZSCH-Geratebau GmbH, Selb, Germany) in the temperature range of 30–600 °C. Approximately 10 mg of sample was placed in Al_2_O_3_ crucible and was analyzed with a heating rate of 10 °C/min under nitrogen atmosphere (flow of purge gas 20 mL/min and protective gas 10 mL/min). A DSC analysis of 5–10 mg of the prepared material was performed on DSC1 (Mettler Toledo, Switzerland) in the temperature range 25–200 °C with a heating rate of 20 °C/min under argon atmosphere (20 mL/min). The heat of evaporation of residual solvents used in the synthesis was determined from the first run of DSC measurement.

The atomic absorption spectrometer (AAS—ContrAA 300, Analytik Jena, Jena, Germany) was used for the measurement of metal ion concentrations in aqueous samples. Zeta potential measurements were conducted using a ZetaSizer Nano ZS (Malvern, UK) to study the surface charge changes of the final materials under different pH. The morphology of the sorbents was studied with an FEI Quanta 250 FEG scanning electron microscope. Images were taken for secondary electrons in the low vacuum mode at a pressure of 70 Pa. An accelerating voltage of 20 kV was used. The XPS analyses were used to confirm which moiety located on the sorbents surface was responsible for the metal adsorption. The analyses were carried out in a PHI VersaProbeII Scanning XPS system using monochromatic Al Kα (1486.6 eV) X-rays focused on a 100 µm spot. The photoelectron take-off angle was 45°, and the pass energy in the analyzer was set to 117.50 eV for survey scans and 46.95 eV to obtain high energy resolution spectra for the C 1s, N 1s, Cl 2p, Br 2p, and O 1s regions. A dual beam charge compensation with 7 eV Ar^+^ ions and 1 eV electrons were used to maintain a constant sample surface potential regardless of the sample conductivity.

### 2.3. Srobents Fabrication

**Figure 1:**
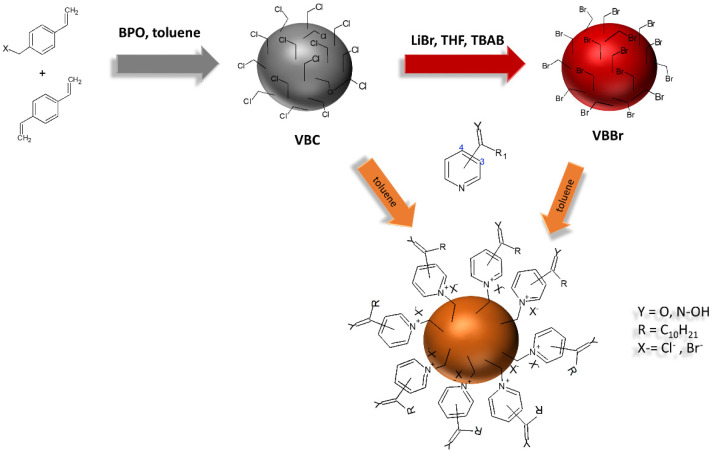
Scheme of the synthesis of the sorbent series VBC and VBBr.

The poly(vinylbenzyl chloride-co-divinylbenzene) (VBC) was prepared according to the procedure described in [32]. After the washing and drying procedure, the VBC beads reacted with the equimolar amount of LiBr in tetrahydrofuran as a diluent. The reaction was supported by the 0.1%mol PTC catalyst (tetrabutylammonium bromide). After finishing the halogen-exchange reaction, the poly(vinylbenzyl bromide-co-divinylbenzene) beads (VBBr) were collected by centrifugation, washed with methanol, and then dried in a vacuum chamber. The fabricated VBC and VBBr beads, after characterization by elemental analysis to determine the amounts of the chloro- and bromomethyl groups located in the polymers structure, were reacted in toluene with the appropriate amount of 1-(4-pyridyl)undecan-1-one (K4.10), 1-(3-pyridyl)undecan-1-one (K3.10), 1-(4-pyridyl)undecan-1-one oxime (Ox4.10), and 1-(3-pyridyl)undecan-1-one oxime (Ox3.10) as precursors of the functional groups. The functionalization was carried out at 50 °C for 7 days in 250 mL glass reactor equipped with a hot-plate–magnetic stirrer. The purified sorbents were dried at 80 °C under vacuum overnight and analyzed (FT-IR, Raman, elemental). The procedure used enabled us to obtain the VBC-K3.10, VBC-K4.10, VBC-Ox3.10, VBC-Ox4.10, VBBr-K3.10, VBBr-K4.10, VBBr-Ox3.10, and VBBr-Ox4.10 sorbents. A scheme of the synthesis of the studied sorbents is presented in Figure 1.

### 2.4. Sorption Studies

Sorption experiments were carried out by employing the batch method by mixing 0.1 g of the sorbent with 100 mL of an aqueous solution of Cu(II), Zn(II), and Cd(II) with variable concentrations of each metal ion at a constant temperature of 23 °C. The adsorption was studied at a different pH (1–6), times (1–350 min), and metal ion concentrations (50–200 mg/L) and at constant temperature (23 °C). The working metal concentrations were prepared by appropriate dilution of stock solutions. The pH of each of the solutions was adjusted to values of 1.0–6.0 by the dropwise addition of 0.1 M NaOH or 0.1 M HCl using the T5 Excellence titrator fitted with pH electrode. The desorption experiment was conducted for the selected sorbents using different aqueous solutions of HCl, NaCl, and Na_2_SO_4_. The mixtures were shaken using a temperature controlled shaker (KS 4000 ic control, IKA) at 240 rpm for a period of desired time. After finishing sorption or desorption, the solid phase was separated and the metal ion concentration in the supernatant was determined by atomic absorption measurements using ContrAA 300 (Analytik Jena AG). Each experiment was replicated three times, and the results were averaged. The metal ion removal efficiency (R) and amount of adsorbed metal ions (Q) were calculated using the following equations:

(1) $$R=\frac{C_{0}-C_{t}}{C_{0}}\cdot 100$$

(2) $$Q=\frac{\left(C_{0}-C_{t}\right)\cdot V}{m}$$

where *C*_0_ and *C_t_* (mg/L) are the concentrations of the metal ions before and after contact between the phases at time *t*. *V* is the volume of aqueous phase used (L), and *m* is the mass of the sorbent used (g).

The experimental data were also used to evaluate the kinetics and equilibrium of the sorption (Table 1). The pseudo-first order, pseudo-second order, Elovich, and intra-particle diffusion models were applied to describe the kinetics of the sorption [33]. The equilibrium was modelled using isotherms such as Freundlich, Langmuir, Dubinin-Radushkevich, and Temkin [34]. The nonlinear forms of the models are presented in [35]. Kinetic studies were carried out using 50 mg/l at pH 5 at 298.15 K. The isotherm experiments were carried out by using different initial concentrations of metal ions (50–200 mg/L) at 298.15 K and at a pH of 5.

## 3. Results and Discussion

### 3.1. Structural and Thermal Characterization of the Sorbents

The FTIR spectra confirmed the fabrication of poly(vinylbenzyl chloride-co-divinylbenzene); the substitution of chlorine atom with bromine; and further quaternization of 1-(4-pyridyl)undecan-1-one (K4.10), 1-(3-pyridyl)undecan-1-one (K3.10), 1-(4-pyridyl)undecan-1-one oxime (Ox4.10), and 1-(3-pyridyl)undecan-1-one oxime (Ox3.10). In the case of VBC, the spectrum confirmed the presence of a –CH_2_Cl functional group located at the benzene ring. The peak at 1261 cm^−1^ was attributed to stretching vibrations of the chloromethyl group, and the peak at 802 cm^−1^ was due to stretching vibrations of C–Cl. The exchange of Cl on Br resulted in a shift of these bands to lower wavenumbers (–CH_2_Br and C–Br vibrations observed at 1071 cm^−1^ and 707 cm^−1^, respectively). Comparing the spectra of VBC or VBBr, and the sorbents after modification (VBC-K3.10, VBC-K4.10, VBC-Ox3.10, VBC-Ox4.10, VBBr-K3.10, VBBr-K4.10, VBBr-Ox3.10, and VBBr-Ox4.10), the peaks at 1600–1605 cm^−1^ and 1508 cm^−1^ assigned to the C=N and C=C ring stretching vibrations of pyridine, respectively, were indicated. Moreover, in the case of the VBC-K3.10, VBC-K4.10, VBBr-K3.10, and VBBr-K4.10 resins, the peaks at 1720, 1722, 1701, and at 1702 cm^−1^, respectively, were assigned to the C=O stretching vibrations of ketone group were observed. In contrast, for VBC-Ox3.10, VBC-Ox4.10, VBBr-Ox3.10, and VBBr-Ox4.10, the bands corresponding to oxime C=N were observed at 1635, 1647, 1632, and 1639 cm^−1^, respectively.

**Figure 2:**
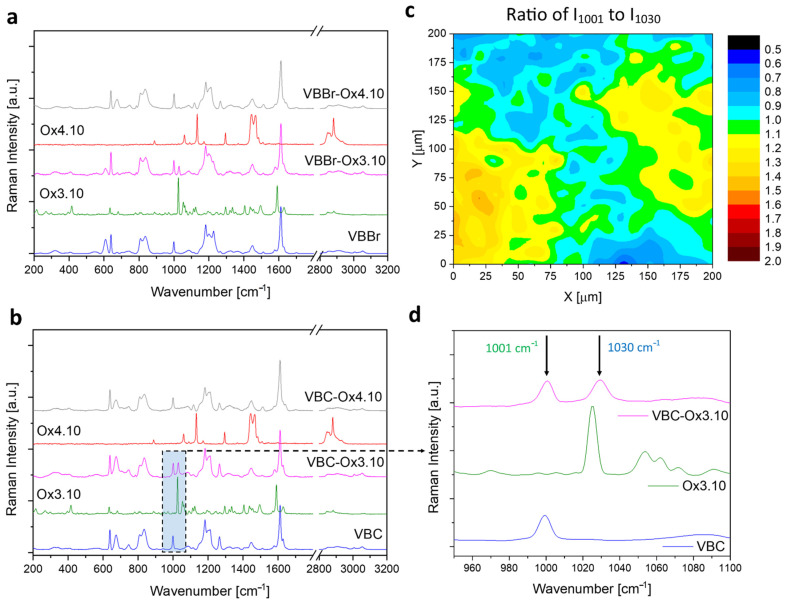
Raman spectra of VBBr (**a**) and VBC (**b**) before and after modification with Ox3.10 and Ox4.10, Raman map of VBC-Ox3.10 (**c**) and Raman spectra with marked bands on the basis of which the map was obtained (**d**).

Obtaining the materials at each stage of the synthesis was also confirmed by Raman analysis (Figure 2a–d). The Raman spectra of VBC and VBBr show differences in the position of the bands. In the spectra of VBC, the bands assigned to the stretching vibrations of –CH_2_Cl and C–Cl are present at 1265 cm^−1^ and 674 cm^−1^, respectively. In turn, in the spectra of VBBr, the bands assigned to –CH_2_Br and C–Br are shifted to lower wavenumbers and are indicated at 1227 and 609 cm^−1^, respectively. In the Raman spectra of all modified VBBr and VBC, there are bands related to VBBr and VBC, and the new bands at 1030 and 1117 cm^−1^ are assigned to C-H vibrations of the pyridine ring in Ox3.10 and Ox4.10, respectively. The Raman map (Figure 2c) presenting the ratio of the 1001 cm^−1^ band intensity (related to VBC) to the 1030 cm^−1^ band intensity (related to Ox3.10) (Figure 2d) was used to evaluate the effectiveness of VBC modification. The Raman map confirms that the VBC was successfully modified. The 1030 cm^−1^ band is observed in all Raman spectra collected on the entire analyzed surface. The other Raman spectroscopy results also confirm that VBC as well as VBBr was efficiently modified with other compounds.

**Figure 3:**
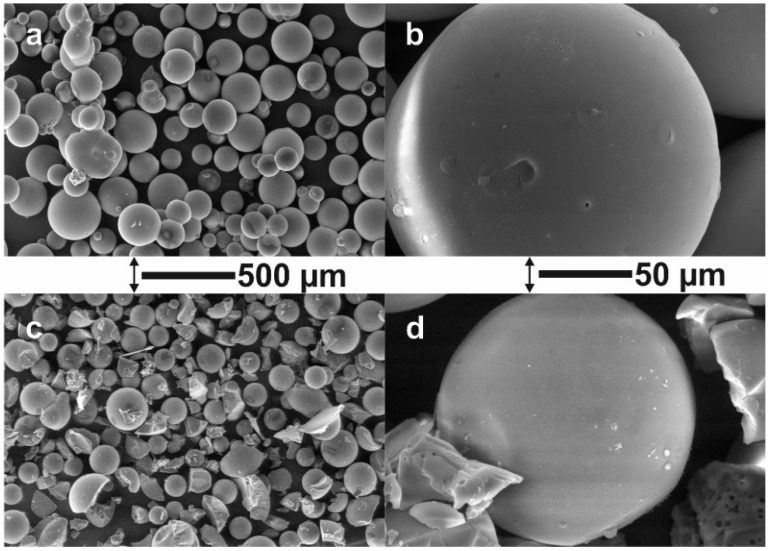
SEM Images of VBC (**a**,**b**) and VBBr (**c**,**d**).

The surface morphologies of VBC and VBBr particles were observed by SEM, and representative images are presented in Figure 3. In the case of VBC, it was observed that more than 90 percent of the imaged grains are in the size range of 175–300 micrometers (diameter). The mean value of the grain diameter is 258 microns, with an average deviation of 46 microns. The shape of the grains is perfectly spherical, and there is an occasional defect that looks similar to a dent from an adjacent ball. Since there is a small population of spheres connected, those with a slight deformation of the shape may be the effect of separation of previously connected balls. The surface of the grains is smooth, and it contains a small number of pores in the form of round holes with a diameter of up to about 1 micrometer. There is also a small population (less than 10%) of very small spheres: 25–75 micrometers. In the case of VBBr, it was indicated that the condition of the substitution reaction caused partial damage to the VBC spheres. Approximately 50% of the beads have been fragmented into several pieces large enough to see areas that were previously the surface of the ball on them. There was no complete decomposition, only cracking. Sizes similar to the undissolved VBC with a mean diameter of 133 micrometers with a mean variation of 31 micrometers and a diameter spread of 90–250 micrometers. The size and shape of the crumbs indicate that the balls break into several or a dozen parts. The pores inside the broken balls are of a spherical shape, constituting a small part of the volume of the spheres. These voids have a diameter of 1 to 5 micrometers. The fractures themselves contain sharp edges, typical for brittle fracture. The surface of undamaged spheres has no holes visible on the VBC. The analysis of the VBC and VBBr after functionalization with the pyridine derivatives did not reveal any further changes in the morphology and shape of the particles.

The measurement of the contact angle provides information on the wettability of water on the sorbent surface. The results obtained show a close correlation between the structure of functional groups and the hydrophilicity of the sorbents (Table S1). The presence of bromide counter ions on the polymer surface causes poor surface wetting (θ > 90°). On the other hand, the surface of the VBC series sorbents shows a contact angle much below 90°, which allows us to classify these materials as hydrophilic. Moreover, substitution on the 4-position of the pyridine ring yields higher contact angle values than that observed for 3-analogue, and the ketones are less hydrophilic than the corresponding oximes.

**Figure 4:**
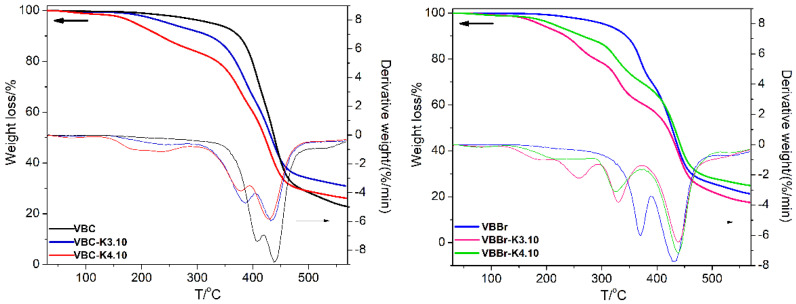
Thermogravimetric curves unmodified and modified VBC and VBBr with K3.10 and K4.10.

The thermal stability of the synthetized materials was examined by thermogravimetric analysis (TGA). Figure 4 shows the TGA curves of two types of copolymers VBC and VBBr, and products of their modification with the appropriate pyridine derivative. Figure 4 shows the weight loss of the materials when heated up to 600 °C under nitrogen atmosphere. As can be seen, copolymers present three steps of decomposition. The first one, occurring at a temperature below 100 °C, can be attributed to the evaporation of the residual solvents used during the synthesis and purification of the sorbents, which are absorbed in samples. The next two are weight losses of copolymer. At a lower temperature range, the decomposition of pendant groups and then decomposition of the crosslinked network of copolymer occur. The initial decomposition temperatures were taken as onset of the weight loss and presented in Table 2. The thermal stability of both copolymers is in the temperature above 300 °C, and the copolymer VBC has greater thermal stability (T_onset_ = 382 °C) than the copolymer with VBBr by approximately 50 °C (T_onset_ = 336 °C). Modification of the copolymers structure causes a decrease in the thermal stability of the starting polymers and the appearance of additional degrees of decomposition on the curves related to the grafted structures. Despite the differences in the thermal resistance of copolymers, their modification results in the obtaining materials with similar resistance to thermal decomposition, which is probably related to the chemical structure of the substituents introduced into the copolymers as a result of the modification. All investigated sorbents are stable up to the temperature of approximately 160 °C, with the Ox3.10 derivatives having the highest stability, i.e., T_onset_ close to 200 °C. Therefore, they are characterized by a higher thermal stability than the commercial Lewatit TP 207 (thermal stability up to 80 °C) or Amberlyst 15 resin (T_onset_ 150 °C).

The research carried out using the differential scanning calorimetry (DSC) confirmed the relationship of the first stage of the decomposition of the synthesized sorbents with the evaporation of the residual solvents. The heat of this transition was calculated from thermograms (example DSC thermogram is shown in Figure S1), and the results are presented in Table 2. The amount of absorbed solvent by the VBBr sorbents increases after modification, while for the VBC sorbents, it practically did not change. Such a solvent absorption may be related to the sorption capacity of the polymer matrix. Characteristic temperatures related to phase transitions of polymers are not observed due to their cross-linked structure.

### 3.2. Sorption Studies

#### 3.2.1. Effect of pH

**Figure 5:**
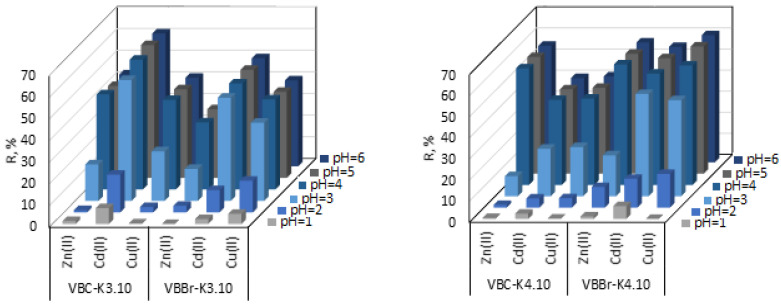

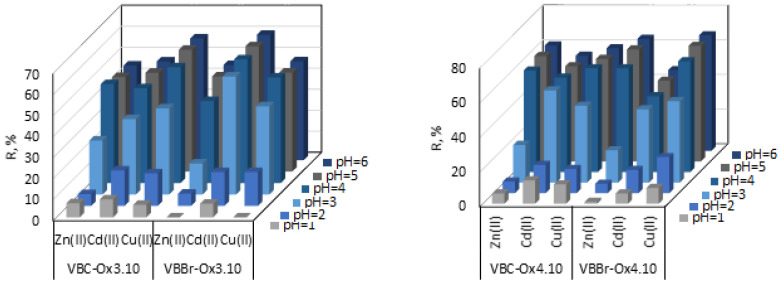
Effect of pH on Pb(II), Cd(II), and Zn(II) sorption with sorbent series VBC and VBBr (M(II) = 50 mg/L, pH = 1–6, time–180 min, sorbent dosage—0.1 g, and V—100 mL).

The pH of the solution is one of the most important parameters influencing adsorption and removal of metal ions from the aqueous feed solution. The percent of metals removal as a function of pH were plotted for all tested metals and illustrated in Figure 5a–d. The presented experimental data show that, regardless of the tested sorbents and the type of metal ions adsorbed, the sorption almost does not occur when the pH is lower than 2. The sorption from solution with pH of 3 is much higher, but only for Cd(II) sorption onto VBC-K3.10 and VBBr-K3.10 as well as for the Cd(II) and Cu(II) sorption onto VBBr-K4.10, VBC-Ox3.10, VBC-Ox4.10, VBBr-Ox3.10, and VBBr-Ox4.10, in which the removal is higher than 50%. Moreover, for the Cd(II) sorption onto VBC-K3.10, VBBr-K3.10, VBBr-K4.10, VBC-Ox3.10, VBC-Ox4.10, VBBr-Ox3.10, and VBBr-Ox4.10, the further increase in the pH value does not significantly affect the sorption efficiency. The most efficient metal ion removal from pH above 4 may be attributed to the fact that the positive species (M^+2^) is the most abundant at this condition and can be attracted by an interaction with the negative zeta potential surface, which is in agreement with the electro-kinetic profile of the sorbents (see Figure S2). From the zeta potential measurements, a positive potential below a pH of 3.0–3.8 was indicated, which changed to a value negative above the isoelectric point.

The sorption results also indicate that the effectiveness of the process depends on the type and the structure of the functional group. For Zn(II) and Cu(II), VBBr-Ox4.10 is much more efficient than other considered materials, while for Cd(II) the VBC-K3.10, resin has been found to be highly effective.

The dependence of the removal efficiency on the pH of the aqueous phase probably is a result of the competitive adsorption effect of the H^+^ ions. This effect and speciation analysis [38] point to sorption of cationic species of Zn(II), Cu(II), and Cd(II) through interactions with the ketone group (through the lone pair of the donor carbonyl oxygen atom interaction) [39] or with the oxime group, which can bind a metal cation via oxygen and nitrogen atoms [40]. To further explore the interactions between metals cations and the appropriate groups, XPS measurement was employed. It was shown that, in the case of the ketone sorbents and the O1s emission, a new low-energy feature at 531.0 eV appeared with the simultaneously decrease in 532.3 eV. The growth of the new feature also resulted in the growth of C 1s emissions at 284.8 and a decrease at 286.4 eV. For VBC and VBBr modified by the oxime derivatives, the appearance of new emission was also observed for N 1s (399.4 eV), and growth emission at 402.26 eV. Both signals were consistent with the presence of –H– and the quaternary N, in the resin structure.

#### 3.2.2. Effect of Time

**Figure 6:**
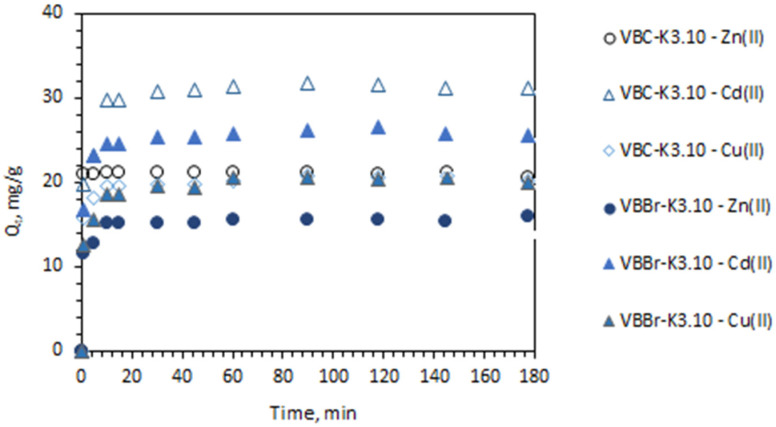
Effect of shaking time on Cu(II), Cd(II), and Zn(II) sorption with VBC-K3.10 and VBBr-K3.10 (M(II) = 50 mg/L, pH = 5, time: 1–180 min, sorbent dosage—0.1 g, and V—100 mL).

The time of contact of sorbents and aqueous feed solutions can influence the efficiency of metals removal. In this study, the effect of contact time was studied by making contact with both phases for 1–180 min. The results obtained indicate that, regardless of the structure of the functionalities, the metal sorption is very fast and equilibrium is reached after 10 min. As shown in Figure 6, the sorption of Zn(II) with VBC-K3.10 is much faster than that observed for other sorbents: 1 min of shaking is enough to achieve equilibrium. In the case of other materials, the results have shown no influence of the functional group structure on the sorption rate.

To analyze the sorption kinetics, the experimental data were fitted by using the selected models (pseudo-first order, pseudo-second order, intra-particle diffusion, and Elovich models). The fitting results such as kinetic rate constants, correlation coefficients, and q_e_ are presented in Table 3. The results unequivocally show that the pseudo-second-order model describes the kinetics. The coefficient of determination value achieved varied from 0.999 to 1.000, and the calculated values of q_e_ were consistent with the experimental results. These results suggest that the rate-determining step is dominated by chemical sorption. In the case of the VBC-K3.10 sorbent, the values of the reaction rate constant k_2_ increase from 0.029, and 0.056 to 1.362 g/mg·min for Zn(II), Cd(II), and Cu(II), respectively. In the case of the VBBr-K3.10 sorbent, the values of k_2_ rise in order Zn(II) > Cd(II) > Cu(II) (0.072, 0.056, and 0.029 g/mg·min, respectively)

#### 3.2.3. Effect of Metals Ions Concentration

**Figure 7:**
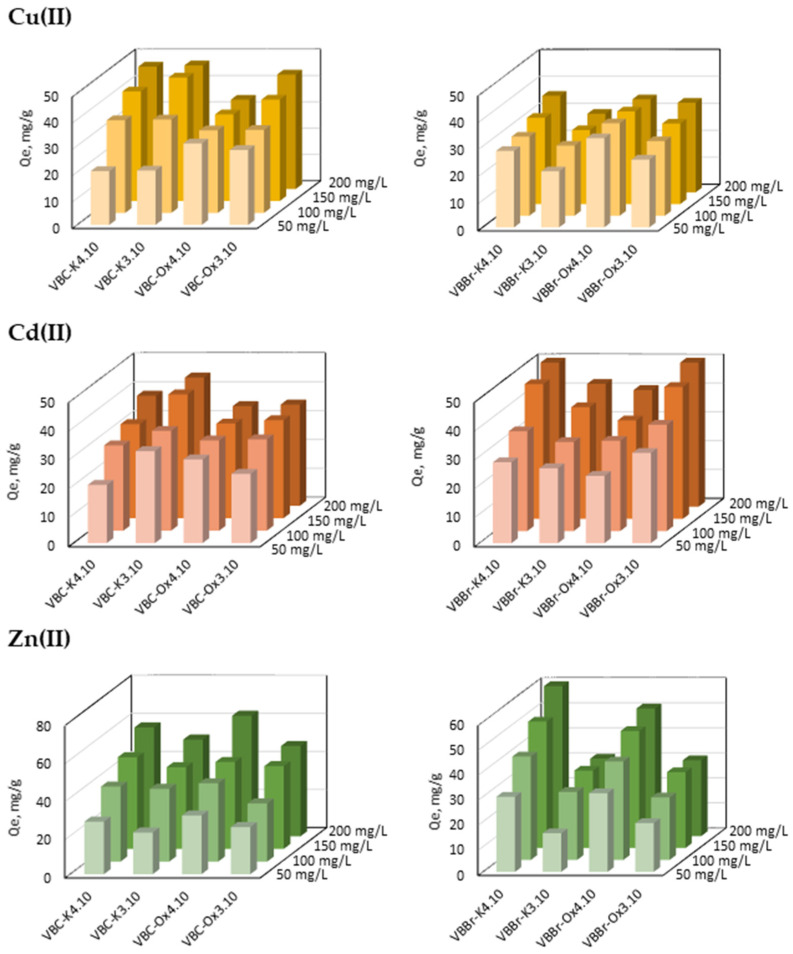
Effect of initial concentration of Cu(II), Cd(II), and Zn(II) on their sorption with VBC and VBBr series sorbents (M(II) = 50–200 mg/L, pH = 5, time: 180 min, sorbent dosage—0.1 g, and V—100 mL).

The influence of the initial metal ion concentration on their removal with the sorbent series VBC and VBBr was also investigated. The experiments were conducted for 3 h of shaking at a pH of 5; at T = 298.15 K; and using the aqueous feed solution containing from 50 to 200 mg/L of Cu(II), Cd(II), and Zn(II). From the results presented ion Figure 7, it can be seen that the removal of metals ions is strongly dependent on their initial concentration in the water phase and on the structure of the pyridinium group located on the sorbents surface.

In the case of the Cu(II) sorption and almost all sorbent series VBC, the metal ions removal increases with the increase in the initial Cu(II) concentration but without saturation achieving. Only for VBC-Ox4.10, the increase is insignificant (Q_e_ increases from 31 to 34 mg/g using the aqueous feed solutions at 50 and 200 mg/L, respectively), which suggests that Q_e_ = 34 mg/g is the maximum loading. An insignificant increase in the Q_e_ value is also observed using VBC-K3.10 as the sorbent and the aqueous feed solutions containing 150 and 200 mg/L of Cu(II). In this case, the results indicate that the value 47 mg/g is slightly off the saturation level. The sorbent series VBBr shows much worse sorption properties towards copper(II). Additionally, the use of aqueous solutions with increasing metal ion concentrations indicates low saturation. The studied sorbents behave differently in contact with the water phase containing cadmium ions. The VBBr series sorbents show much better sorption properties than the VBC series sorbents. For example, 1 g of VBBr-K4.10 removes 52 mg of Cd(II), while VBC-K4.10 only removes 38 mg. A similar effect is observed for VBBr-Ox3.10 and VBCl-Ox3.10 (53 and 35 mg/g, respectively). Moreover, in the case of the VBC sorbents, only VBC-K3.10 removes more Cd(II) than 40 mg/g.

The sorption of Zn(II) did not show more significant differences between the sorbents of the VBC and the VBBr series than that observed for Cu(II) and Cd(II). In the case of the series VBC, zinc(II) can be removed in 63 mg/g (VBC-Ox4.10), while in the case of the VBBr series, zinc(II) can be removed in 60 mg/g (VBBr-K4.10). However, comparing the structure of the functional group located on the surface of the VBC or VBBr matrix, the differences can be observed. The modification with K4.10 was much better with VBBr-K4.10, while the modification with K3.10 was much better with VBC-K3.10. Am opposite relationship can be observed when comparing sorbents VBC-Ox3.10 and VBBr-Ox3.10 as well as VBC-Ox4.10 and VBBr-Ox4.10. The sorbent series VBC is much more efficient than the sorbent series VBBr.

The above experimental data were also fitted to four isothermal models: Freundlich, Langmuir, Dubinin–Radushkevich, and Temkin. Modelling is necessary for the determination of the sorption mechanism and to predict the effectiveness of the sorption system. The isotherm parameters are summarized in Tables S2 and S3. The correlation coefficients indicate that the Langmuir model fits the data better than other considered models (for the VBC series, R^2^ varies from 0.995 to 1.000, while for the VBBr series, the R^2^ values vary from 0.992 to 0.999). Fitting the Langmuir isotherm model indicates that sorption takes place on a homogeneous surface as a result of complexation (coordination or ion exchange mechanisms). In such a process, metal ions are adsorbed in the form of a monolayer and no interactions between the adsorbate are observed. The equilibrium constant R_L_ has also been calculated, and for almost all tested sorbents, the values for each metals sorption are between 0 and 1, indicating the adsorption as favorable. Only for the Cu(II) sorption onto the VBBr-Ox4.10 surface is R_L_ equal 0, which indicates an irreversible process. A very good fit has also been noted for the Temkin model (R^2^ = 0.991–0.998), especially in the case of the Cu(II) and Zn(II) sorption onto VBC-K3.10; the Cu(II), Cd(II), and Zn(II) sorption onto VBC-K4.10; the Cd(II) sorption onto VBBr-K4.10, VBBr-Ox3.10, and VBBr-Ox4.10; as well as the Zn(II) sorption onto VBC-Ox4.10. Fitting the Temkin isotherm model also confirms that the adsorption of the demonstrated systems follows a chemisorption process.

The estimated adsorption abilities were also compared with the sorption properties of strongly acidic (Amberlite IR 120) and weakly acidic (Lewatit CNP 80) ion-exchange resins as well as with the latest tested composites poly (ethyleneimine)-silica gels and poly (amidoamine)-graphene oxide and with xanthan-modified magnetic chitosan (see Table 4). It can be seen that, compared with most of the sorbents mentioned, VBC-K3.10 has a greater adsorption capacity in relation to Cu(II), Cd(II), and Zn(II). Only Amberlite IR 120 shows much stronger properties in relation to Cd(II). The strong adsorption capacity of Amberlite IR 120 can be attributed to its strong interaction and to the process being carried out at a pH above 6, which for Cu(II), Zn(II), and Cd(II) resulted not only in the metal–resin interaction but also in the precipitation of hydroxides.

#### 3.2.4. Effect of Temperature

**Figure 8:**
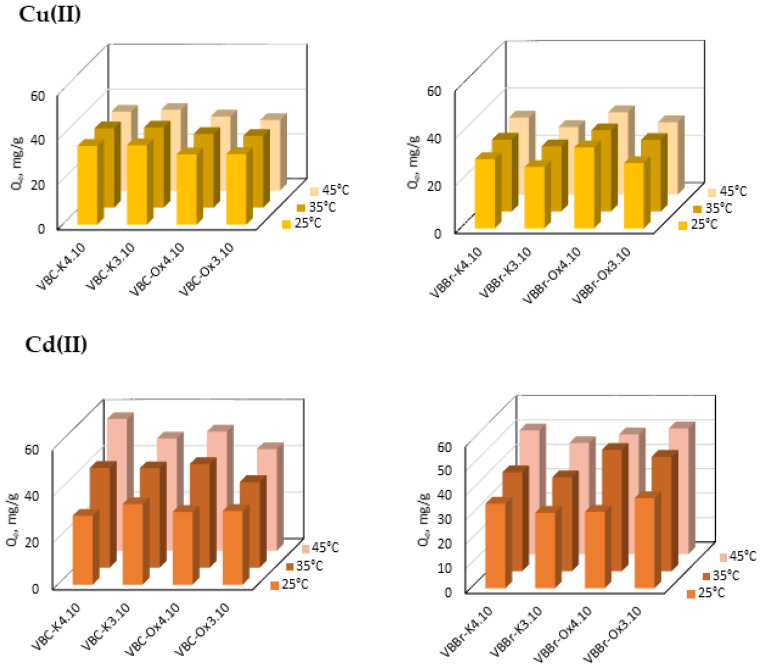

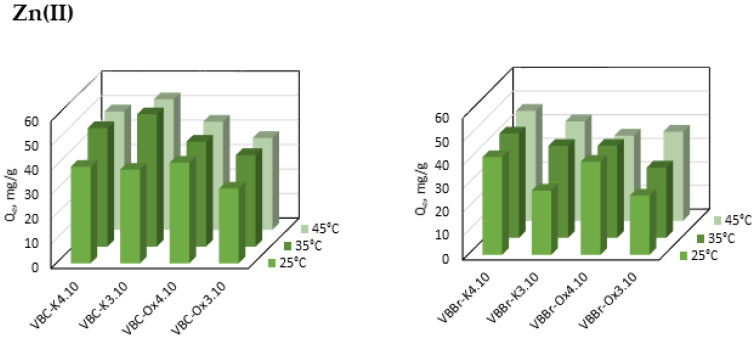
Effect of temperature on Cu(II), Cd(II), and Zn(II) sorption with VBC and VBBr series sorbents (M(II) = 100 mg/L, pH = 5, time: 180 min, sorbent dosage—0.1 g, and V—100 mL).

The temperature at which the sorption process is carried out can influence the removal of the metals ions. Therefore, in these studies, that effect was also considered. A series of experiments were performed at temperatures of 25, 35 and 45 °C; at a constant pH value of 5; and at the constant metal ion concentration of 100 mg/L. The results obtained are presented in Figure 8.

From the presented results, it can be concluded that the sorption of Cu(II) does not change remarkably with the change in temperature. A similar effect is observed for the Zn(II) sorption with the sorbents obtained by the modification with Ox4.10. In contrast, in the case of the Cd(II) sorption and other Zn(II)-VBC and Zn(II)-VBBr systems, the increase in temperature makes the sorbents more efficient. The effect is significant when using the VBC and VBBr series materials as sorbents of Cd(II). For instance, the sorption of Cd(II) with VBC-K3.10 increases by almost 100% if the temperature is changed from 25 to 45 °C. In the case of the zinc(II) sorption, the effect is particularly evident using the VBBr-K3.10 and VBBr-Ox3.10 resins, which were found as the least efficient sorbents of Zn(II). The increase in the sorption may result from the increased diffusion rate of the adsorbate ions through the surface boundary layer, especially if the hydrophobic nature of the surface was found. This increase may also be due to changes in the availability of the active sites capable of coordinating with the metal ions. Here, the temperature can cause isomerization changes in the oxime C=N or conformational changes in the decile chain.

### 3.3. Reusability Test

The sorbent application potential is influenced not only by the sorption capacity or the kinetics of the process but also by the desorption of the adsorbed component with the participation of relatively mild reagents. Therefore, experiments were also carried out in order to develop the most optimal desorbing agent. This agent not only should easily desorb the metal ions but also should enable sorption immediately after regeneration in subsequent cycles of the process. After sorption (100 mg/L Cu(II), Cd(II), or Zn(II) at a pH of 5 and at a temperature of 45 °C), the 1 g of sorbents (VBC-K3.10 and VBBr-K3.10) were contacted with 10 mL of 0.1 and 0.01 M HCl and with a 0.5 M solution of NaCl and Na_2_SO_4_. Immediately after desorption, the separated sorbent was contacted with the fresh aqueous feed solution. The sorption–desorption cycle was repeated five times.

The obtained results indicated that, regardless of the sorbent used and the metals ions removed, the desorption of the metals with the salts solutions was not very efficient (desorption with the NaCl solution ranged from 41 to 59%, while using the Na_2_SO_4_ solution, it ranged from 52 to 64%). Slightly better results were obtained using 0.01 M HCl (58–72%), but the 0.1 M HCl solution was found to be the most efficient desorbing agent. The results also showed that five-fold repetition of the sorption–desorption procedure using the 0.1M HCl solution as the desorbing agent did not adversely affect the adsorption properties of VBC-K3.10 and VBBr-K3.10 (the differences in the removal efficiency were 5–7% and 4–8%, respectively).

## 4. Conclusions

Based on the conducted research, it was shown that the proposed procedure of the copolymer of vinylbenzyl chloride and divinylbenzene modification through quaternization in toluene as well as halogen exchange according the S_N_2 mechanism (poly(vinylbenzyl bromide-co-divinylbenzene) synthesis) enabled us to obtain novel ionic liquid-imprinted polymer sorbents. The sorption studies clearly show that the sorption properties depended not only on the type of metals ions and on the functional group structure (ketone or oxime substituent located at 3- or 4-position in pyridine ring as well as chloride or bromide counter ion for pyridinium nitrogen) but also on the pH, temperature, and initial concentration of metal ions. Both pH as well as temperature sorption increases with the increase in values of the parameters. The pH dependence indicated the sorption of cationic forms of metals, which at a pH below 2 competed with free H^+^. This effect also indicated the ease of desorption, e.g., with diluted acid. The temperature effect indicated that the process was exothermic or that the increased temperature increased the availability of the active centers capable of coordinating to the metals ions. This indicated, in conjunction with thermogravimetric analysis, a high sorption potential of the resins at higher temperatures than 25 °C. The results obtained also showed that, regardless of the structure of the functionalities, the metal sorption was very fast and equilibrium was reached even within 1 min of shaking. The fitting to the selected isotherm models indicated that sorption takes place on the homogeneous surface as a result of complexation (coordination by the imine moiety or the oxime OH). Among the selected compounds, there was none that turned out to be the most effective for all absorbed metal ions, e.g., for Zn(II) sorption, the most efficient was VBC-Ox4.10 (84 mg/g); for Cd(II) sorption, the most efficient was VBBr-Ox3.10 (63 mg/g); and for Cu(II) sorption, the most efficient was VBC-K3.10 (69 mg/g). Moreover, the possibility of reusing the sorbent allowed us to state that these materials could be successfully used for Cu(II), Cd(II), and Zn(II) removal.

## Figures and Tables

**Table 1:** Kinetic and isotherm models with equations used to describe sorption process.

Kinetic Model	Equation *	Equation
Pseudo-first order	$q_{t}={\;q}_{e}\;\left(1-e^{-k_{1}t}\right)$	(3)
Pseudo-second order	$q_{t}=\frac{k_{2}\cdot q_{e}^{2}}{1+k_{2}\cdot q_{e}}$	(4)
Elovich	$q_{t}=\frac{1}{\beta }\ln\left(\alpha \beta t\right)$	(5)
Intra-particle diffusion	$q_{t}={\;k}_{\mathrm{ip}}t^{\frac{1}{2}}+C$	(6)
Isotherm model	Equation **	
Langmuir	$q_{e}=\frac{q_{m}K_{L}C_{e}}{1+K_{L}C_{e}}$	(7)
Freundlich	$q_{e}={\;K}_{F}{\;C}_{e}^{\frac{1}{n}}$	(8)
Dubinin-Radushkevich	$q_{e}={\;q}_{m}e^{-K_{\mathrm{DR}}\;{\left[\mathrm{RT}\;\ln\left(1+\frac{1}{C_{e}}\right)\right]}^{2}}$	(9)
Temkin	$q_{e}=\frac{\mathrm{RT}}{b}\ln(K_{T}C_{e})$	(10)

* q_e_ and q_t_ (mg/g) are the amount of metal ions removed at equilibrium and at time t (min), k_1_ the pseudo-first-order model (min^−1^), k_2_ (g/mg min) is the pseudo-second order rate constant, k_ip_ is the intra-particle diffusion rate constant (mg/g min^0.5^), α (mg/g min) is the initial sorption rate constant, and β (g/mg) is the desorption constant. ** C_e_ is the equilibrium concentration of adsorbed metal ions. K_L_ (L/mg), K_F_ (mg^1-(1/n)^/gL^n^), K_DR_ (mol^2^/J^2^), and K_T_ (L/g) are the appropriate constants. q_m_ is the maximum sorption capacity, and n is the heterogeneity factor.

**Table 2:** Thermal properties of sorbents (and for comparison commercial resins).

	T_onset_, °C	Hp**, J/g	Tp**, °C	Ref.
VBC	382.3	23.1	83.9	
VBC-K3.10	185.4	12.4	88.3	
VBC-K4.10	159.7	26.3	100.9	
VBC-Ox3.10	193.8	26.1	93.2	
VBC-Ox4.10	179.4	27.6	90.2	
VBBr	336.4	10.1	80.0	
VBBr-K3.10	158.2	36.6	88.0	
VBBr-K4.10	182.1	45.6	90.2	
VBBr-Ox3.10	195.5	16.1	77.2	
VBBr-Ox4.10	191.8	25.2	85.3	
Lewatit TP 207	80			[36]
Amberlyst 15	150			[37]

T_onset_—temperature of decomposition of polymers determined from TG measurements. Hp**—heat of solvent evaporation determined from DSC thermograms. Tp**—the temperature at the maximum of heat evaporation peak determined from DSC thermograms.

**Table 3:** Kinetic parameters of Cu(II), Cd(II), and Zn(II) sorption with VBC-K3.10 and VBBr-K3.10.

Kinetic Model	VBC-K3.10			VBBr-K3.10		
	Cu(II)	Cd(II)	Zn(II)	Cu(II)	Cd(II)	Zn(II)
q_exp_ (mg/g)	20.8	31.9	21.4	20.9	26.2	15.9
Pseudo-first order						
k_1(_min^−1^)	0.028	0.035	0.015	0.029	0.033	0.023
q_e.cal_ (mg/g)	20.8	31.9	21.3	20.9	26.2	15.9
R^2^	0.915	0.913	0.667	0.954	0.804	0.854
Pseudo-second order						
k_2_ (g/mg min)	0.056	0.029	1.362	0.032	0.056	0.072
q_e_ (mg/g)	20.7	32.1	21.4	20.8	26.2	15.9
R^2^	0.999	0.999	1.00	0.999	0.999	0.999
Intra-particle diffusion						
k_ip2_ (mg/g min^0.5^)	1.18	2.12	0.99	1.37	1.61	0.95
C	10.9	14.2	13.6	9.1	12.9	7.9
R^2^	0.763	0.723	0.997	0.781	0.604	0.997
Elovich						
α (mg/g min)	106.3	61.7	660.5	39.3	76.4	59.32
β (g/mg)	0.376	0.208	0.450	0.325	0.271	0.469
R^2^	0.954	0.925	0.926	0.965	0.863	0.926

**Table 4:** Comparison of capacities towards Cu(II), Cd(II), and Zn(II) of the selected adsorbents.

Adsorbent	Adsorption Capacity, mg/g			Ref.
	Cu(II)	Cd(II)	Zn(II)	
Amberlite IR 120	21.9	101.0	85.0	[41]
Lewatit CNP 80	10.2	4.9	20.3	[42]
poly(ethyleneimine)-silica gels	38.5	-	52.1	[43]
poly(amidoamine)-graphene oxide	8.7	-	13.2	[43]
xanthate-modified magnetic chitosan	34.5	55.0	20.8	[44,45]
VBC-K3.10	68.6	47.6	72.3	This work

## Data Availability

Not applicable.

## Supplementary Materials

The following are available online at https://www.mdpi.com/article/10.3390/ma14175008/s1, Figure S1: DSC thermogram of the VBBr-Ox3.10 sorbent, Figure S2: Values of the zeta potential measured as a function of pH, Table S1. Values of the isoelectric point and contact angle of fabricated sorbent series VBC and VBBr, Table S2: Isotherms parameters of different models for the sorption of Cu(II), Zn(II), and Cd(II) onto VBC-K3.10, VBBr-K3.10, VBC-K4.10, and VBBr-K4.10, Table S3: The isotherm parameters of different models for the sorption of Cu(II), Zn(II), and Cd(II) onto VBC-Ox3.10, VBBr-Ox3.10, VBC-Ox4.10, and VBBr-Ox4.10.
