# Supplementary material for: Novel Polymer Sorbents with Imprinted Task-Specific Ionic Liquids for Metal Removal
Source: Materials (Basel). 2021 Sep 2;14(17):5008. doi: 10.3390/ma14175008 (PMC8434268; doi:10.3390/ma14175008)
Supplement: Supplementary file 1 [file materials-14-05008-s001.zip › materials-1335780-supplementary.pdf]

materials

Supplementary Materials

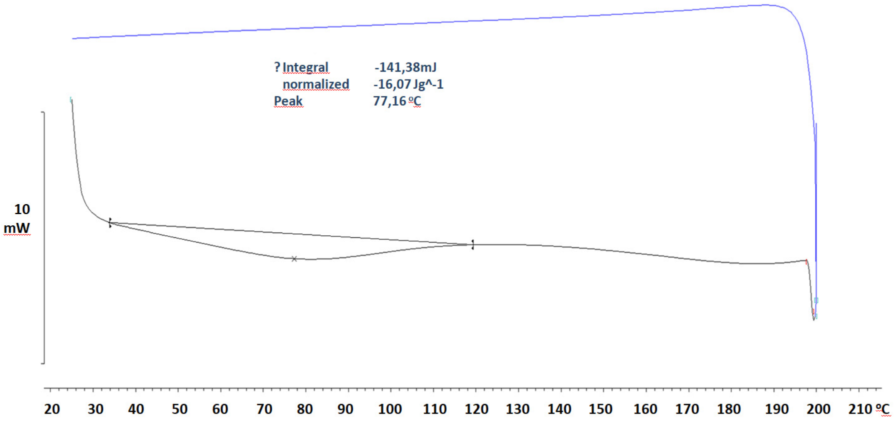

Figure S1. DSC thermogram of VBBr-Ox3.10 sorbent.

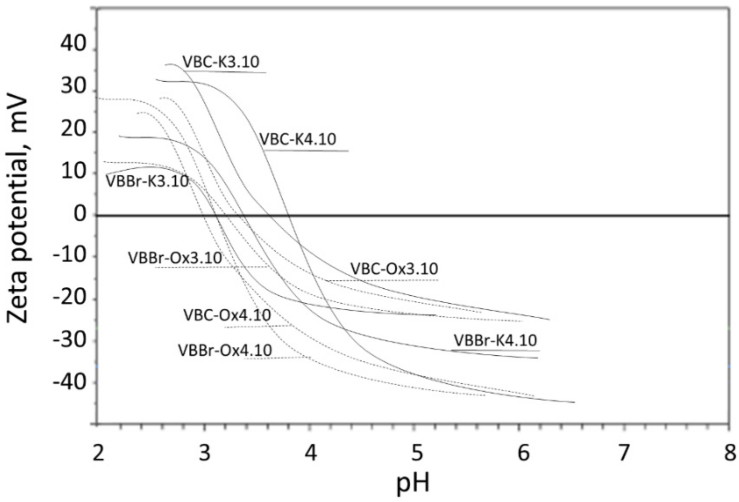

Figure S2. Values of zeta potential measured as a function of pH

Table S1. Values of isoelectric point and contact angle of fabricated sorbents series VBC and VBBr

	IEP	Contact angle (degree)	Ref.
VBC-K3.10	3.6	72.2	
VBC-K4.10	3.8	77.1	
VBC-Ox3.10	3.3	63.9	
VBC-Ox4.10	3.0	74.8	
VBBr-K3.10	3.1	122.4	
VBBr-K4.10	3.1	124.3	
VBBr-Ox3.10	3.2	93.9	
VBBr-Ox4.10	3.1	98.4	

Table *S2*. Isotherms parameters of different models for sorption of Cu(II), Zn(II) and Cd(II) onto VBC - K3.10, VBBr - K3.10, VBC – K4.10, VBBr – K4.10

**Isotherm model**	**VBC - K3.10**			**VBBr - K3.10**			**VBC - K4.10**			**VBBr - K4.10**		
	Cu(II)	Cd(II)	Zn(II)	Cu(II)	Cd(II)	Zn(II)	Cu(II)	Cd(II)	Zn(II)	Cu(II)	Cd(II)	Zn(II)
Langmuir												
Q_m (mg/g)	68.6	47.6	72.3	31.6	49.0	37.1	66.3	47.2	73.1	37.7	60.2	75.3
K_L (L/mg)	0.01	0.08	0.02	0.06	0.04	0.03	0.02	0.02	0.02	0.08	0.04	0.03
R_L	0.7	0.2	0.5	0.3	0.3	0.4	0.5	0.5	0.5	0.2	0.3	0.4
R^2	0.997	0.998	0.997	0.992	0.997	0.999	0.999	0.996	0.995	0.997	0.998	0.996
Freundlich												
K_F ($mg^{1-(1/n)}/gL^n$)	3.76	19.72	4.23	11.12	10.95	6.25	3.87	5.81	7.29	17.90	10.68	8.91
n	1.91	6.22	1.99	5.28	3.72	3.07	1.99	2.69	2.38	7.66	3.14	2.56
R^2	0.976	0.718	0.986	0.976	0.913	0.924	0.973	0.988	0.985	0.796	0.991	0.992
Dubinin-Radushkevich												
Q_m(mg/g)	44.5	41.9	47.2	28.2	39.5	31.1	44.3	35.7	52.9	33.4	48.0	55.0
K_{DR} (mol²/kJ²)	$1.1\cdot10^{-4}$	$1.6\cdot10^{-5}$	$9.8\cdot10^{-5}$	$4.5\cdot10^{-5}$	$4.1\cdot10^{-5}$	$8.2\cdot10^{-5}$	$1.2\cdot10^{-4}$	$8.4\cdot10^{-5}$	$6.1\cdot10^{-5}$	$1.8\cdot10^{-5}$	$4.4\cdot10^{-5}$	$4.6\cdot10^{-5}$
R^2	0.903	0.786	0.899	0.940	0.786	0.963	0.931	0.897	0.896	0.683	0.892	0.8411
Temkin												
K_T (L/g)	0.12	9.72	0.13	3.14	0.70	0.35	0.13	0.24	0.21	32.68	0.46	0.27
B	151.5	417.4	149.9	532.9	281.0	324.0	156.0	243.1	154.0	611.9	207.1	154.5
R^2	0.995	0.978	0.991	0.985	0.976	0.948	0.992	0.991	0.994	0.934	0.999	0.989

Table *S3*. Isotherms parameters of different models for sorption of Cu(II), Zn(II) and Cd(II) onto VBC-Ox3.10, VBBr-Ox3.10, VBC–Ox4.10, VBBr–Ox4.10

**Isotherm model**	**VBC-Ox3.10**			**VBBr-Ox3.10**			**VBC-Ox4.10**			**VBBr-Ox4.10**		
	Cu(II)	Cd(II)	Zn(II)	Cu(II)	Cd(II)	Zn(II)	Cu(II)	Cd(II)	Zn(II)	Cu(II)	Cd(II)	Zn(II)
Langmuir												
Q_m (mg/g)	47.9	38.3	58.8	35.2	63.0	35.4	34.5	35.5	84.2	34.6	49.2	56.6
K_L (L/mg)	0.05	0.07	0.03	0.08	0.04	0.04	0.23	0.14	0.02	1.18	0.03	0.05
$\mathbf{R}_L$	0.3	0.2	0.4	0.2	0.3	0.3	0.1	0.1	0.5	0.0	0.4	0.3
R^2	0.995	1.000	0.996	0.998	0.999	0.995	0.999	0.998	0.998	0.995	0.999	0.996
Freundlich												
K_F ($mg^{1-(1/n)}/gL^n$)	14.7	12.4	7.6	15.8	11.9	7.7	27.2	22.5	7.0	31.4	7.2	15.9
n	4.73	4.77	2.71	7.04	3.29	3.63	24.88	12.37	2.21	52.77	2.90	4.32
R^2	0.874	0.953	0.980	0.885	0.981	0.948	0.793	0.968	0.997	0.933	0.983	0.987
Dubinin-Radushkevich												
Q_m(mg/g)	40.0	34.5	44.2	31.3	49.9	29.7	32.9	33.1	57.2	34.4	38.0	46.8
K_{DR} (mol²/kJ²)	$2.8\cdot10^{-5}$	$4.06\cdot10^{-5}$	$5.99\cdot10^{-5}$	$2.38\cdot10^{-5}$	$3.8\cdot10^{-5}$	$6.3\cdot10^{-5}$	$3.8\cdot10^{-6}$	$9.8\cdot10^{-6}$	$5.7\cdot10^{-5}$	$2.0\cdot10^{-6}$	$6.8\cdot10^{-5}$	$2.2\cdot10^{-5}$
R^2	0.751	0.981	0.860	0.779	0.916	0.868	0.478	0.760	0.909	0.985	0.879	0.776
Temkin												
K_T (L/g)	1.98	2.17	0.27	17.74	0.57	0.58	$9.0\cdot10^{8}$	$3.8\cdot10^{3}$	0.18	$1.9\cdot10^{21}$	0.30	1.49
B	334.8	406.4	193.7	607.9	205.4	364.9	1908.6	990.7	133.0	3877.5	241.0	276.2
R^2	0.969	0.968	0.990	0.975	0.997	0.950	0.787	0.957	0.996	0.935	0.998	0.971

materials

MDPI

d on Day Month Year).
